# A novel computational approach for the mining of signature pathways using species co-occurrence networks in gut microbiomes

**DOI:** 10.1186/s12866-024-03633-6

**Published:** 2024-11-21

**Authors:** Suyeon Kim, Ishwor Thapa, Hesham Ali

**Affiliations:** College of Information Science and Technology, University of Nebraska at Omaha, Omaha, NE 68182 USA

**Keywords:** Microbial signature, Microbial co-occurrence network, Network analysis, Enriched pathways comparison

## Abstract

**Background:**

Advances in metagenome sequencing data continue to enable new methods for analyzing biological systems. When handling microbial profile data, metagenome sequencing has proven to be far more comprehensive than traditional methods such as 16s rRNA data, which rely on partial sequences. Microbial community profiling can be used to obtain key biological insights that pave the way for more accurate understanding of complex systems that are critical for advancing biomedical research and healthcare. However, such attempts have mostly used partial or incomplete data to accurately capture those associations.

**Methods:**

This study introduces a novel computational approach for the identification of co-occurring microbial communities using the abundance and functional roles of species-level microbiome data. The proposed approach is then used to identify signature pathways associated with inflammatory bowel disease (IBD). Furthermore, we developed a computational pipeline to identify microbial species co-occurrences from metagenome data at various granularity levels.

**Results:**

When comparing the IBD group to a control group, we show that certain co-occurring communities of species are enriched for potential pathways. We also show that the identified co-occurring microbial species operate as a community to facilitate pathway enrichment.

**Conclusions:**

The obtained findings suggest that the proposed network model, along with the computational pipeline, provide a valuable analytical tool to analyze complex biological systems and extract pathway signatures that can be used to diagnose certain health conditions.

**Supplementary Information:**

The online version contains supplementary material available at 10.1186/s12866-024-03633-6.

## Background

In the past two decades, new biomedical technologies have made it possible to obtain high-throughput biological data for many health conditions. Given the availability of such new data, it’s a natural step for biomedical researchers to conduct new studies to obtain new biological insights that can be used for classification purposes, prediction of clinical outcomes of interest, and responses to therapy. Recent advances in high throughput data have revolutionized the way we understand the role of microbiomes in various environments, including the microbiome communities in the human body.

The potential for microbiome-based interventions increases as we improve our understanding of microbiome associated health conditions [1–3]. The characterization of microbial roles in host health and disease is therefore essential for achieving non-invasive, personalized, and accelerated treatment options. The growing evidence for differences in the gut microbiome of people with undesirable health conditions compared to healthy controls suggests that certain changes in the microbiome can be served as a new microbiome signature to characterize diseased groups. Guo et al. have revealed significant differences of microbiome abundance, microbial species interactions and functional pathways in Myalgic encephalomyelitis/chronic fatigue syndrome (ME/CFS) subjects and healthy controls [4, 5]. Lee et al. have demonstrated the microbial signatures that are associated with two major colorectal cancer (CRC) precursor lesions [6]. It is also worth noting that these microbial species may also be linked to environmental factors, such as diet and medications [6].

As a response to these recent developments, recently developed bioinformatics tools such as the HMP Unified Metabolic Analysis Network (HUMAnN) are now widely used to generate taxonomic, functional, and strain-level profiles from raw metagenomic sequencing data [7]. While the output of such tools can be directly interpreted with basic downstream analyses, they may not be ideal for distinguishing microbial community profiles, in terms of composition and inter-relationships, in differentiating disease states from healthy states. To this end, shotgun metagenomic sequencing data are now publicly available and can be readily obtained from multiple independent studies. Pooling such datasets, while challenging, provides a valuable source of information that can be used for identifying robust and reproducible microbial signatures that are consistent across many different studies.

Metagenomic-based studies currently provide valuable insights into the composition of the microbiome in both healthy individuals and those diagnosed with certain diseases. Several research studies emphasize the importance of understanding potential associations among microbial species, as these bacterial communities interact with each other within the host [8–10]. Although identifying such microbial occurrence patterns is complicated, these patterns of microbial co-occurrence might provide insights to their biological implications. Despite recent progress in microbiome research, limited progress has been made to identify specific microbial patterns associated with host health phenotypes.

This is mainly due to microbial heterogeneity and diverse functional roles. Current computational tools largely focus on the identification of differentially abundant microbes from abundance data [11, 12]. In addition, microbiome network-based analysis, including NetCoMi [13] and iNAP [14] have been proposed. For example, NetCoMi (Network construction and comparison for microbiome data) is an R package that provides functions for the construction and analysis of microbial networks that are associated with certain phenotypes. One of the major challenges associated with these efforts is that different groups in microbial communities may have similar biological functions, especially evidenced in healthy individuals [15, 16]. However, current efforts for the identification of signature pathways based on co-occurring microbial species are rare. Also, certain groups of microbial communities may play different roles to support different biological functions.

In this study, we propose a new computational approach to identify microbiome based functional enrichment patterns associated with host disease states. The pipeline focuses on mining a high-level summary of the enriched pathways underlying microbial communities associated with host health and disease states. This pipeline is designed to allow researchers to analyze microbial communities at different granularity levels: a single microbe, microbial sub-communities, or the overall microbial network structures. When comparing microbial communities in healthy versus disease states, it is important to address several research questions: What bacterial species are central in these communities? How do community networks differ across phenotypes? How conserved are these communities across datasets obtained from different studies? What subgroups of bacterial species (or clusters) in these communities are functionally enriched in a specific disease state?

The proposed pipeline offers multiple functionalities for researchers to construct and analyze species co-occurrence networks as well as functional enrichment analysis. This make it possible to identify which single species, groups of species, or the overall network of species contribute to enriched pathways associated with health-related conditions. While this study is focused on samples for inflammatory bowel disease (IBD) groups, the proposed computational pipeline can be used to analyze the datasets associated with other types of diseases and health conditions. In this study, we used metagenome samples for two IBD groups, consisting of Crohn’s disease (CD) and ulcerative colitis (UC) from two independent studies.

## Methods

Due to many confounding factors, the role of a microbial community associated with a specific host phenotype is not reproducible across multiple datasets. To overcome this challenge and identify microbial species and communities associated with pathways conserved across different datasets, we introduce a novel computational pipeline that contains 4 major steps: (A) Construct a species co-occurrence network from filtered samples; (B) Analyze the species co-occurrence network at three granularity levels (single microbe, microbial sub-community, and overall microbial network structure); (C) Perform pathway enrichment analysis; (D) Compare enriched pathways between two IBD datasets in each sample from healthy/disease states.

**Fig. 1:**
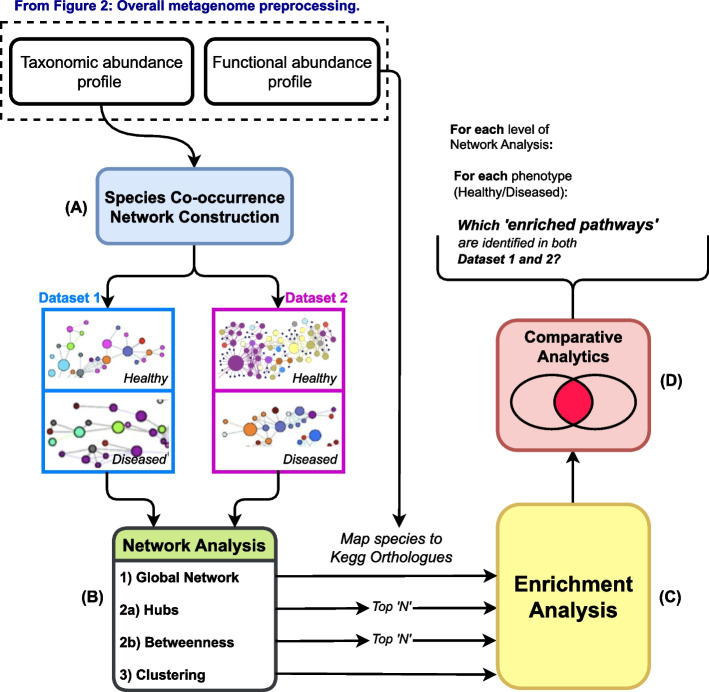
Novel computational pipeline for mining signature pathways that are associated with host health conditions: **A** Construction of a species co-occurrence network using taxonomic profiles; **B** Microbial species co-occurrence network analysis; **C** Enrichment analysis; **D** Identification of robust enriched pathway. The two required inputs are pre-processed before using the pipeline. Detailed steps for pre-processing data are provided in Fig. 2

To achieve this, we combine enriched pathways across the aforementioned three analyses to identify robust enriched pathways with contributing species, thereby indicating possible functional markers associated with IBDs. An overview of the computational pipeline is shown in Fig. 1.

### Data pre-processing

As a case study to show the utility of this computational pipeline, we obtained two publicly available human stool metagenome datasets and their metadata. Raw metagenomic sequences of two published inflammatory bowel disease (IBD) studies were obtained from NCBI Sequenced Read Archive PRJNA398089 [17] and PRJNA400072 [18].

**Sample selection criteria**: Samples were removed based on several exclusion criteria. We excluded samples pertaining to medication interventions (i.e., antibiotics, immunosuppressant) and participants who were younger than 18 or older than 65 years of age. Furthermore, duplicate sample IDs were removed.

**Fig. 2:**
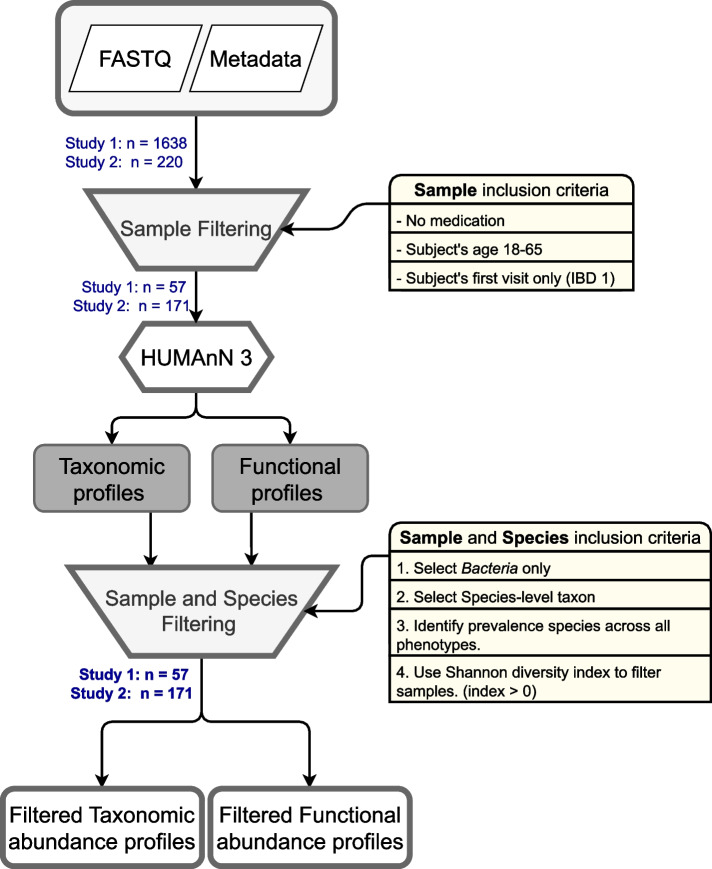
Flowchart depicting a microbial species profile from multiple studies filtering process. Rounded rectangles indicate inputs and outputs. A hexagon indicates a process using a software tool and trapezoids indicate sample and species filtering processes

The study from Franzosa et al. [18] employed cross-sectional data from subjects. Since the study from Lloyd-Price et al. [17] employed longitudinal sampling of subjects, only samples from the subject’s initial doctor visit were included. After this process, the number of samples in study 1 changed from 62 to 57 (see Fig. 2).

#### Profiling the abundance of microbiome and other molecular functions

Metagenomic samples were taken from two IBD cohorts containing Crohn’s disease (CD) and ulcerative colitis (UC), as well as non-IBD controls. Analysis was performed using HUMAnN [7]. A summary of sample sizes after data pre-processing is shown in Table 1.

**Table 1:** Overview of the number of samples used in the study

**Dataset 1**			
non-IBD controls	CD	UC	Total
13	26	18	57
**Dataset 2**			
non-IBD controls	CD	UC	Total
54	69	48	171

MetaPhlAn version 3.1.0, which can provide pan-microbial (bacterial, archaeal, viral, and eukaryotic) profiling, was used to generate taxonomic profiles of shotgun metagenomes from 57 and 171 samples in dataset 1 and dataset 2, respectively. Functional profiling was performed by HUMAnN version 3.1.1. The output of this tool includes gene-families abundance profiles (UniRef90s), which can be summarized as KEGG Orthologues (KOs).

#### Species and sample filtering based on taxonomic profiles

After taxonomic profiling, we chose to exclude archaea and eukaryote due to their low prevalence in the majority samples. We also removed zero prevalance species across non-IBD control, CD, and UC phenotypes. The zero prevalence species are those that are not present in any of the samples. Note that the focus of this study is to construct species-level co-occurrence, as the species level taxon provides comprehensive microbiome information. With the species abundance data, we measured Shannon diversity index to calculate species diversity and richness using the R ‘vegan’ package [19]. Shannon diversity index is a widely used method for measuring biological diversity [20]. Samples with zero Shannon diversity index were excluded. A schematic overview of metagenome pre-processing is shown in Fig. 2.

### Creating the species co-occurrence network

After pre-processsing the species-level abundance and subsequent filtration of species and samples, the species relative abundance matrix was converted into a species presence/absence matrix. Abundance values greater than zero were considered “present”. The resulting binary matrix in which each row is a species and each column is a sample represents presence or absence of a species in a sample. Based on this matrix, co-occurrence probabilities at the species level were measured in each phenotype. To accomplish this, we utilized the probabilistic model of species co-occurrence from the ‘*cooccur*’ R package [21]. The co-occurrence probabilities for each species pair were retrieved as a table containing the pair information, observed number of samples having both species, and the probability of both species occurring in samples from a phenotype. The significance levels of positive and negative co-occurrence patterns were obtained by measuring how greater or lesser the probabilities of those co-occurring species were than the observed frequencies. Then, from all to all species pairs, we extracted significantly positive co-occurrence pairs with $`p\_gt' < 0.05$. For the significant positive species pairs, we constructed the unweighted species co-occurrence network, where the species are represented as nodes and edges are drawn if two species are significantly co-occurring in that phenotype within each dataset.

### Network analysis

Next we studied different network properties in these co-occurrence networks from all phenotypes in two datasets. Network properties can be evaluated at multiple granularity levels. For example, an element level with a focus on a node or an edge, community level with a focus on a group of nodes and edges, and finally the complete networks. In this study, we examined the species co-occurrence networks at all three levels. These three levels of analyses were performed on species co-occurrence networks from each phenotype in both IBD datasets as described below:

#### Global network analysis

We analyzed the overall species co-occurrence network from the non-IBD control, CD, and UC groups. For each group, we performed functional enrichment analysis based on the total number of species captured from these co-occurrence networks. Enriched pathways for each diseased group were compared against the non-IBD control. We evaluated the pathways that were consistently enriched across multiple datasets.

#### Community-level analysis

To identify groups of species that were highly connected between each other, we performed the Leiden community detection algorithm to detect species-communities in the networks from each phenotype. We used the Constant Potts Model (CPM) as the objective function. The value of the resolution parameter is based on the ratio of the quartile value of strength of the nodes and the total number of nodes as shown in the igraph package in R [22]. Functional enrichment of each cluster (community) of bacterial species was compared with the notion that bacterial communities work together to achieve a function. Across two datasets, we identified the functions that were consistently enriched within the same phenotype.

#### Key element-level analysis

Basic descriptive network properties of the species co-occurrence networks in non-IBD control, CD, and UC were analyzed using the *igraph* R package [22]. We measured centrality of the network to measure topological importance of nodes within the network. Specifically, hub and betweenness centrality were quantified. ‘Hub’ measures the degree of connectedness of a node to all other nodes within a network and can be inferred as how central the node is based on the eigenvector centrality. ‘Betweenness centrality’ of a node is a measurement of how many shortest paths within the network pass through the node, which is critical to maintaining the connectedness of that network. We obtained the top 5 species nodes with hub and betweenness centrality, respectively. In this case, the selection of threshold did not impact on the result. Further, we characterized the roles of these central nodes by performing functional enrichment analysis and comparing those across datasets.

### Functional enrichment analysis

**Mapping Species-to-KO:** The obtained list of species from the aforementioned analyses from three levels were then mapped to their KEGG Ortholog identifiers (KO). This mapping step requires KO abundance profiles generated by the HUMAnN pipeline. The abundance profiles from the HUMAnN pipeline contain abundances of KOs stratified by each species and an aggregate abundance value for that KO. We mapped our species of interest to KOs that they contribute to. Next, this list of KOs was used to perform pathway enrichment analysis.

**Pathway enrichment analysis:** After mapping species with their KO information, we performed KEGG pathway enrichment analysis with the *enrichKO* function in the *MicrobiomeProfiler* package in R [23]. This function performs hypergeometric test to find enrichment for each pathway by utilizing the number of KOs from our species of interest, the KOs annotated for a pathway and all the KOs in the background (universe). The result table contains the pathway information, gene ratio, adjusted *p*-value, and geneIDs for each pathway.

In addition, for each dataset and at different network level analyses, we compared the corresponding lists of enriched pathways between the disease phenotype and healthy phenotype.

Subsequently, the pathways unique to disease conditions were compared across two datasets. These identified unique pathways across datasets indicate possible functional markers associated with the disease phenotype. We visualized those identified common pathways associated with diseased phenotypes to determine which genes from the co-occurring species contributed to those pathways. We generated this pathway of interest graph by specifying the list of gene IDs (KO-IDs) and pathway ID using the ‘*pathview*’ function [24].

## Results

### Global comparison of co-occurrence networks in different health conditions

The total number of significant positive species co-occurrences from non-IBD control, CD, and UC are shown in Table 2. Co-occurrence networks in dataset 2 are larger than in dataset 1 across phenotypes. Between different phenotypes, the co-occurrence network from CD is always bigger than non-IBD in both datasets.

**Table 2:** Summary of significant co-occurrence of species for non-IBD control, CD, and UC in each dataset

	Dataset 1			Dataset 2		
	non-IBD controls	CD	UC	non-IBD controls	CD	UC
*p*-value < 0.05	522	721	329	2743	3722	3987

#### Pathways enriched in global species co-occurrence networks for diseased groups across datasets

After pathway enrichment, we compared the enriched pathways in diseased groups (CD and UC) against the non-IBD control and identified common pathways across two IBD datasets (as shown in Table 3). With a significance less than 0.05, the *ABC transporter pathway (map02010)* was enriched in the co-occurring species from CD in both datasets. Similarly, the *Cysteine and methionine metabolism pathway (map00270)* was common to UC in both datasets. The total number of co-occurring species for those enriched pathways in CD and UC are described in Table 3.

**Table 3:** Result of common enriched pathways in CD and UC from global co-occurrence network

Phenotype	Enriched pathways	No. of co-occurring species
CD	ABC transporters (map02010)	165
UC	Cysteine and methionine metabolism (map00270)	144

### Enriched pathways based on community species

The co-occurring species in a community may indicate similar functional capability. We investigated this through clustering and pathway enrichment analysis. For each co-occurrence network, we extracted clusters and identified the membership of the clusters. In CD, we detected 10 clusters in IBD dataset 1 and 55 clusters in IBD dataset 2. UC also contained 7 and 61 clusters in these two datasets, respectively. Then, pathway enrichment of each cluster of species was compared across the datasets. We found 3 enriched pathways for CD and 1 enriched pathway for UC, showing that these pathways were consistently identified between two IBD datasets. We also summarized the number of co-occurring species for each cluster that were found in the enriched pathways associated with diseased groups (as shown in Table 4). We found that the sulfur relay system (map04122) was enriched in multiple clusters for both CD and UC. We also found that differences in enriched pathways distinguished CD: *ABC transporter (map02010)* and *Two-component system (map02020)*. Note that the ABC transporter pathway was also enriched in global species co-occurrence networks in CD. Overall, these results showed that these co-occurring species in each cluster are highly enriched for distinct pathways for CD and UC, suggesting these species need further study.

**Table 4:** Table showing the commonly enriched pathways for each diseased group across two IBD datasets. For each commonly enriched pathway, the cluster that was enriched *(Cluster No.)*, the total number of co-occurring species in that cluster (*Total Species*) and the species that specifically contributed to the pathway (*Contrib. Species*) are shown

	Pathways	Dataset 1			Dataset 2		
		*Cluster no.*	*Total species*	*Contrib. species*	*Cluster no.*	*Total species*	*Contrib. species*
CD	ABC transporter (map02010)	CL 5	15	10	CL 2	223	129
	Two-component system (map02020)	CL 5	15	7	CL 2	223	119
	Sulfur relay system (map04122)	CL 5	15	4	CL 54	1	1
UC	Sulfur relay system (map04122)	CL 4	4	3	CL 57	1	1

**Fig. 3:**
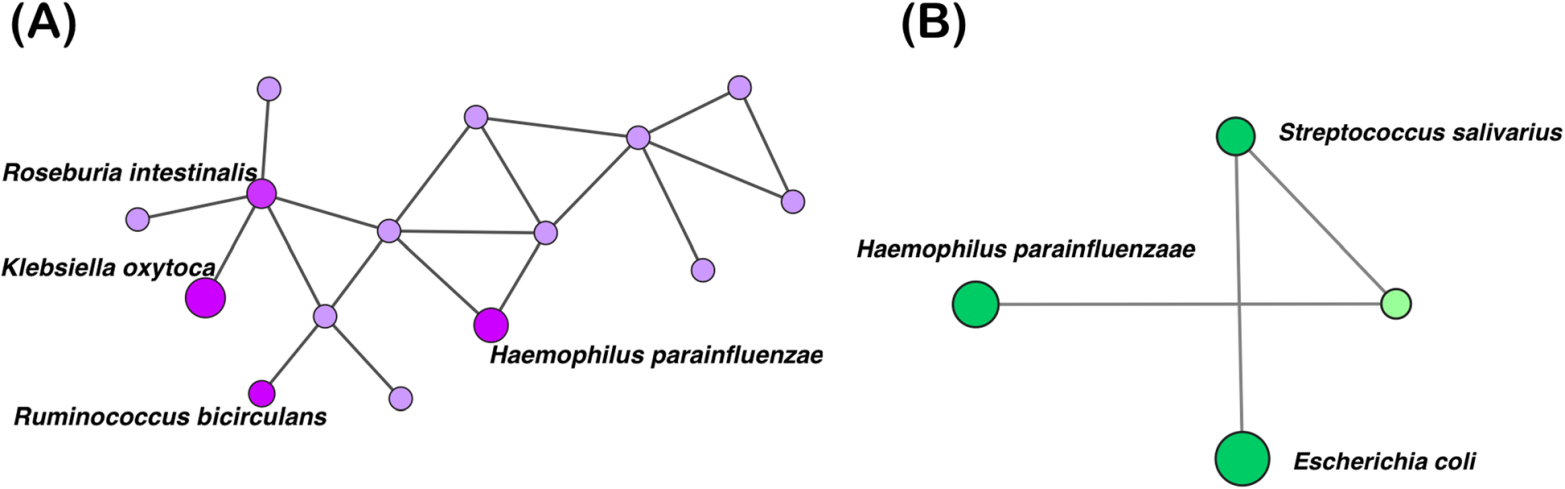
Clusters in CD and UC in IBD dataset1 enriched in sulfur relay system pathway as shown in Table 4. The networks show the microbial co-occurring communities in Cluster No. 5 from CD group (A:Left) and Cluster No. 4 from UC group (B:Right). Nodes are colored by their diseased group (purple indicates CD and green indicates UC). The size of the nodes corresponds to the number of KOs each bacteria contributes to in the sulfur relay pathway. Nodes are colored brighter when the co-occurrence species are contributing to this pathway

**Table 5:** List of co-occurring species and count of KOs contributing to the *sulfur relay system* pathway

Dataset	Phenotype	Name of species	Number of KOs
Dataset 1	CD	*Haemophilus parainfluenzae*	9/29
		*Klebsiella oxytoca*	18/29
		*Roseburia intestinalis*	6/29
		*Ruminococcus bicirculans*	1/29
	UC	*Escherichia coli*	15/29
		*Haemophilus parainfluenzae*	9/29
		*Streptococcus salivarius*	1/29
Dataset 2	CD	*Haemophilus sp HMSC71H05*	8/29
	UC	*Haemophilus sp HMSC71H05*	8/29

Figure 3 presents a visualization of clusters identified as enriched in the sulfur relay pathways in both CD and UC from dataset 1. In Fig. 3A, a total of 15 bacterial species were populated in cluster 5 and KOs from 4 species, highlighted in a brighter color, were enriched for the sulfur relay pathway. For UC, we found 4 bacterial species present in cluster 4. Among these 4, KOs from 3 species were enriched in the sulfur relay pathways (shown in Fig. 3B).

### Co-occurring species feature sulfur relay system pathway

**Fig. 4:**
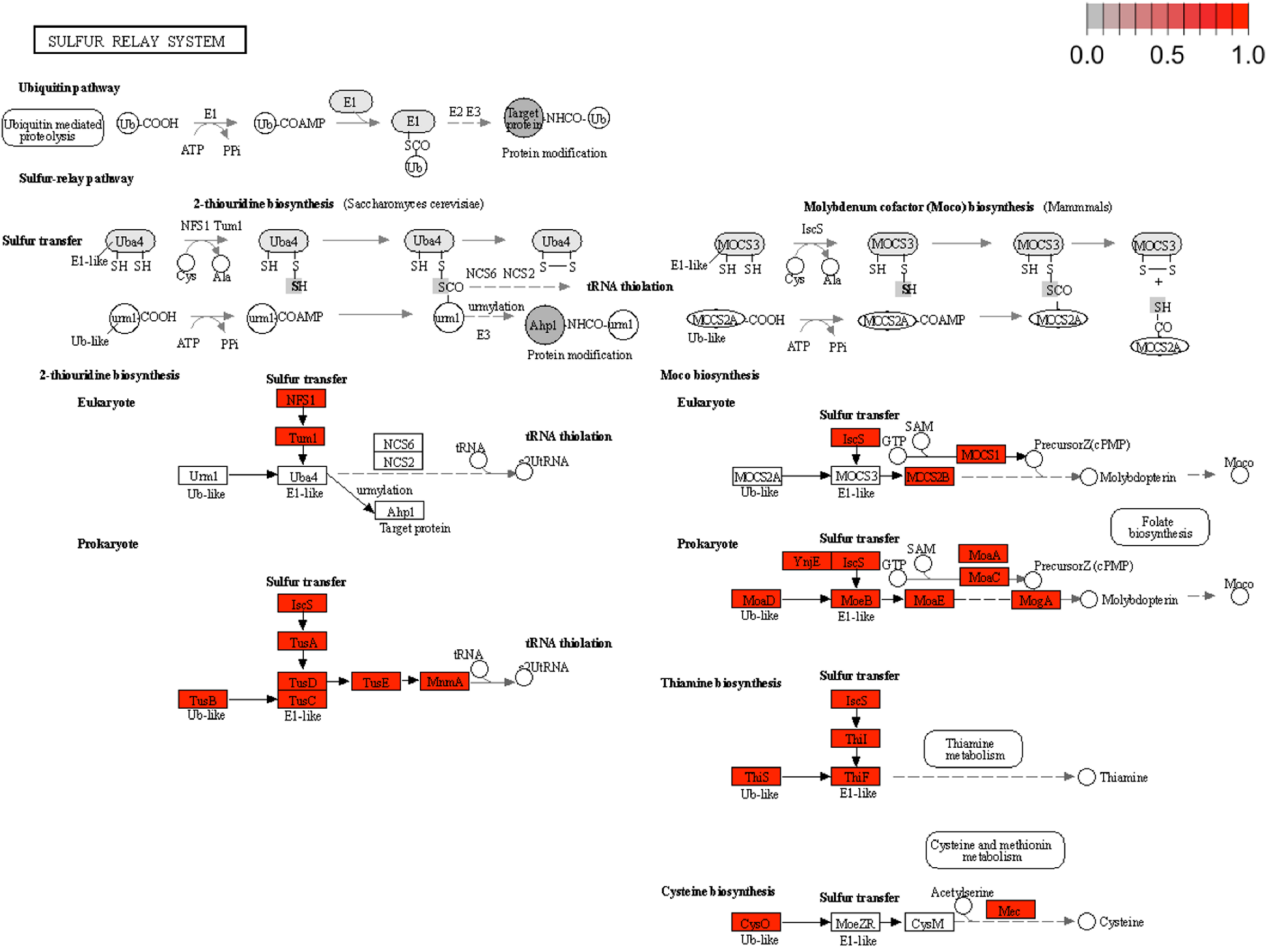
Sulfur relay system pathway with KOs(gene) from co-occurring species in CD. KOs from co-occurring species are highlighted in the color red

Additionally, we visualized the pathway graphs in order to investigate which co-occurring species’s KOs (genes) are contributing to the commonly enriched pathway (Fig. 4). For example, the sulfur relay system (map04122) pathway from CD was further analyzed with the KOs attributable to species (highlighted in red) (see Fig. 4). A total of 29 genes are encoding the sulfur relay system pathway while 20 genes were identified in co-occurring species (these genes were highlighted). The pathway map figure for the sulfur relay system pathway from UC was also included as a figure in Additional file 2 (see Fig. S5).

The co-occurring species counts for the sulfur relay system pathway cluster in CD and UC between two datasets, respectively, are shown in Table 4. For the sulfur relay system pathway, 4 co-occurring species in CD 1 and 1 species in CD 2. Similarly, 3 co-occurring species in UC 1 and 1 species in UC 2. The *Haemophilus parainfluenzae* and *Haemophilus sp HMSC71H05* species were identified in both CD and UC. The *Klebsiella oxytoca* species has the most number of KOs in this pathway.

We quantified the presence of KOs in each species (see Table 5) and found that our identified community of bacterial species have KOs, contributing to a majority of the prokaryotic sulfur relay system pathway. Note that not a single bacterium is able to encode KOs to complete this pathway by itself.

### Enriched pathways based on centrality measures

We identified KOs from the top 5 species by two centrality measures (hub and betweenness) for enrichment in CD and UC from both IBD datasets. Enriched pathways for CD and UC were compared to identify pathways that are consistently enriched in each phentoype across datasets. We compared enrichment results for these top 5 species and their KOs for CD and UC against non-IBD controls. We also found that list of species with betweenness centrality for the UC in dataset 1 (*Alistipes shahii, Alistipes finegoldii, Oscillibacter sp 57 20, Collinsella aerofaciens,* and *Ruminococcus torques*) are different from dataset 2 (*Asaccharobacter celatus, Clostridium leptum, Eubacterium siraeum, Firmicutes bacterium CAG 103,* and *Ruminococcus torques*). The common enriched pathways for the top 5 hub species from CD and UC in two datasets are shown in Table 6. For CD, we observed the top 5 species *Alistipes shahii*, *Bacteroides thetaiotaomicron*, *Coprococcus comes*, *Odoribacter splanchnicus*, and *Parabacteroides merdae* based on top hub scores in dataset 1 and also in dataset 2, including *Alistipes finegoldii*, *Asaccharobacter celatus*, *Blautia obeum*, *Eubacterium hallii*, and *Lachnospiraceae bacterium OF09 33XD*. The most enriched pathways for CD were *pyrimidine metabolism (map0024)* and *streptomycin biosynthesis (map00521)*, which was not identified in UC. For UC, *lysine biosynthesis (map00300)* and *peptidoglycan biosynthesis (map00550)* pathways were the uniquely enriched pathways. Similarly, we also examined the top 5 species with high betweenness centrality in CD and UC co-occurrence networks. Enrichment results for CD and UC versus non-IBD controls are shown in Table 7. While we found 4 enriched pathways in the UC, no enriched pathways were found in CD.

**Table 6:** Result of common enriched metabolic pathways in CD and UC based on Top 5 Hub nodes

Phenotype	Enriched pathways based on Top 5 Hub
CD	Biosynthesis of cofactors (map01240)
	Cell cycle - Caulobacter (map04112)
	Homologous recombination (map03440)
	Mismatch repair (map03430)
	Protein export (map03060)
	Pyrimidine metabolism (map00240)
	Streptomycin biosynthesis (map00521)
UC	2-Oxocarboxylic acid metabolism (map01210)
	Cell cycle - Caulobacter (map04112)
	Drug metabolism - other enzymes (map00983)
	Homologous recombination (map03440)
	Lysine biosynthesis (map00300)
	Mismatch repair (map03430)
	One carbon pool by folate (map00670)
	Peptidoglycan biosynthesis (map00550)

**Table 7:** Result of common enriched metabolic pathways in CD and UC based on Top 5 Betweenness nodes

Phenotype	Enriched pathways based on Top 5 Betweenness
CD	None
UC	Homologous recombination (map03440)
	Mismatch repair (map03430)
	Protein export (map03060)
	Valine, leucine and isoleucine biosynthesis (map00290)

## Discussion

In this work, we propose a computational approach to identify a microbiome-based functional enrichment pattern in host disease states. The main goal of the proposed approach is to identify microbial communities that are collectively working to achieve specific biological functions. It has been reported in several studies that co-occurring microbial species with high frequency may be indicative of biological functions governing community structure [25, 26]. Once microbial communities are identified, the pathway enrichment analysis further reveals their biological significance and highlights which pathways these co-occurring bacterial species contribute to. Since there is little overlap between microbial co-occurrence networks across datasets, augmenting species co-occurrence networks with functional-level analysis to identify health/disease associated signatures is needed.

While numerous network inference methods to infer microbial co-occurrences from microbiome abundance datasets are available, it is still difficult to identify true microbial co-occurrences within a complex microbial ecosystem. Therefore, we performed another inference method, SPIEC-EASI [27] to infer the species co-occurrence network and compare it to the probabilistic approach using the ‘cooccur’ R package. We found that not all highly inferred microbial species interactions with SPIEC-EASI were captured using ‘cooccur’.

The runtime for constructing the network using SPIEC-EASI was significantly better, taking only 3 minutes and 38.664 seconds, in contrast to ‘cooccur’, which took 230 minutes and 26.225 seconds. However, upon manual inspection of some of the highly inferred interactions from SPIEC-EASI, we observed that their abundance values were almost zero across all samples in that phenotype and were still highly correlated (see Additional file 1). Due to the spurious correlations identified in this approach, we believe the probabilistic method from ‘cooccur’ is more suitable in this case.

We analyzed the network properties in these co-occurrence networks at multiple granularity levels. Interestingly, we found that most of the central nodes (based on hub and betweenness measures) appeared in only one of the highly connected clusters obtained from the community level analysis (see Additional file 2). We note that different pathways from functional enrichment were significant at different granularity levels. However, all of these pathways were relevant to IBD. This suggests that analysis at multiple granularity levels provided a unique opportunity to identify significant pathways relevant to IBD.

As seen in Table 2, we observed that there were more significant co-occurrences of species in the second dataset. This could be due to the larger number of samples in this study. We first compared the similarity of the species co-occurrence networks for three phenotypes (CD-CD, UC-UC, and control-control) across datasets. Jaccard index was used to determine how many of the species in the network for dataset 1 overlapped with that of dataset 2. For each phenotype, we expected the Jaccard index score for each comparison to be high; however, this was not the case. The Jaccard index scores for control-control, CD-CD and UC-UC were 0.27, 0.42, and 0.32, respectively. Furthermore, Zelezniak et al. [8] noted that if communities involve phylogenetically related species, then they are likely to be functionally dependent. For example, if ‘Genus A - Species B’ is observed in dataset 1 and ‘Genus A - Species C’ is detected in dataset 2, the Species C in dataset 2 may be functionally similar to Species B in dataset 1.

Given this knowledge, co-occurring species pairs may not be reproducible across independent datasets. However, species level analysis can be useful to understand biological insights in depth. Therefore, we focus on pathway-based signatures between the IBD dataset rather than the species signatures. The two enriched pathways in each diseased group at the global network analysis are shown in Table 3. *ABC transporters (map02010)*, an enriched pathway in CD, is one of the membrane transporters involved in the uptake of a variety of small molecules such as nutrients, ions, and drugs [28, 29]. Previous studies have shown associations between transport alterations and IBD pathophysiology [30–32]. Transporter expression is frequently downregulated in IBD, suggesting that the regulation of ABC transporters may allow for the management and treatment of IBD. Similarly, the Cysteine and methionine metabolism pathway was enriched in UC. Increased excretion of amino acids such as cysteine and methionine has been used to explain why pediatric IBD patients metabolize protein differently than healthy patients [33].

We observed that multiple genes from species under each cluster are collectively enriched in several pathways in CD and UC across datasets as shown in Fig. 3. *ABC transporter* and *Two-component system *(TCSs) pathways were only consistently enriched in CD. The KEGG pathway map of these two pathways are also included in Additional file 2 (Fig. S3 and S4). ABC transporter, as previously noted, have described their alterations in IBD pathophysiology through multiple studies. The TCSs pathway is a signaling mechanisms, ubiquitously present in bacteria and involved in transcriptional regulatory activities such as metabolism and chemotaxis [34, 35]. Shwa et al. described that TCSs regulate bacterial virulence in response to the interaction between host and the transcriptional regulation of bacterial genes in the gut [35].

Similarly, the sulfur relay system pathway was enriched in both CD and UC. The association between the sulfur relay system (map04122) pathway and IBD groups (both CD and UC) was found in several studies [36, 37]. Ni et al. observed that the sulfur relay system provides sulfur for biosynthesis of Molybdenum cofactor (Moco) and thiamin, which are sulfur-containing cofactors [36]. As seen in Fig. 4, various genes are involved in the synthesis of molybdenum cofactor, catalyzing redox reactions in the bacterial metabolism of sulfur. In Table 7, we found that no single bacterial species has all 29 KOs as annotated by KEGG for the sulfur relay system pathway. This highlights the need for the microbial community to work together to perform a common function. Our findings suggest that combining co-occurring species networks with pathway enrichment analysis can identify signature pathways and underlying microbial communities associated with certain health conditions.

Measuring network centrality allows researchers to identify key species that may have important roles within the microbial community. We identified these central nodes with top hub scores and high betweenness centrality. Even though the top 5 list of CD hub-species for dataset 1 is different from dataset 2, these datasets share commonly enriched pathways. We see similar patterns with the top 5 list of UC hub-species in datasets 1 and 2, where the datasets share commonly enriched pathways. As seen in Tables 6 and 7, we were able to identify several consistently enriched pathways in CD and UC for both datasets. Interestingly, we found pyrimidine metabolism and streptomycin biosynthesis only appears in CD. Fernandes et al. found that pyrimidine metabolism was significantly enriched in CD and performed additional validation to identify individual metabolic pathways that are associated with CD through an untargeted metabolomics approach [38]. We also measured the top 5 list of species with betweenness centrality. We found that valine, leucine and isoleucine biosynthesis is particularly enriched in UC species that have high betweenness centrality. Jagt et al. found that the most differentiating features were increased levels of valine and leucine in IBD versus non-IBD controls [39]. Specifically, the authors observed that the abundance of valine was significantly higher in UC patients.

Our work provides a way to bolster the extraction of microbial species co-occurring analysis with pathway analysis. We augmented microbiome datasets from independent IBD studies to identify co-occurring species in a reliable way. Although the pipeline is agnostic to the number of samples, we utilized case studies that included a bigger dataset as well as a medium size one. The number of samples between the case versus and control is somehow unbalanced, however the proposed pipeline can identify key functional pathways for case vs. control comparison within a given dataset and across datasets, which in some ways does overcome the unbalanced number of samples across and within datasets.

Therefore, adding more independent IBD studies would be a natural step for future studies to add robustness to the results we presented. There are a handful of current challenges analyzing the complex systems using metagenome data by itself. The shotgun metagenome sequencing approach allows researchers to investigate microbial profiles in a sample as a community as well as their potential functional profiles. However, this poses a limitation for the identification of microbial genes that are expressed in the samples. To overcome this challenge, our study can be expanded to include analysis of co-occurring species in metatranscriptome datasets.

Overall, our pipeline captures the phenotype-relevant pathways through multi-granularity network analysis from the metagenome abundance datasets. We highlight the functions of microbial communities that form a complex microbial ecosystem, which is based on the species-level microbial associations. We also introduce a computational approach for microbiome researchers to easily implement functional-level analysis of microbial co-occurring communities.

## Conclusion

As we get access to more microbiome-relevant data, we gain more opportunities to establish associations between the profiles of a gut microbiome and their health related implications. While establishing such associations based only on the composition of the microbiome may not always be possible due to the diverse nature of the species forming microbiomes and the roles they can play, we can establish strong associations via their functional behavior and the associated signature pathways. This study demonstrates the viability of identifying and characterizing pathways enriched by highly co-robial species for subjects with specific phenotypes. To illustrate how such characterizing pathways can be used as biological signals, we use datasets collected from subjects with CD and UC. We developed a computational pipeline specifically designed and implemented to identify these co-occurring species and their associated pathways in disease phenotypes. The computational pipeline takes advantages of different types of available biological data, utilized graph and complex network models, and employs different algorithmic techniques and data analytic tools to achieve its goal.

Using the developed pipeline, we were able to identify enriched pathways associated with the diseased groups that can be used for classification or early recognition of the diseased groups. We believe that the obtained results are important since they open new doors to obtain a deeper understanding of the functional roles in the microbial community. The study also suggests that further studies of microbiomes may pave the way to non-invasive methods to diagnose certain health conditions at their early stages as well as to better assess the progress of various treatment options.

## Supplementary information

Supplementary Material 1.

Supplementary Material 2.

## Data Availability

The datasets supporting the conclusions of this article are available in NCBI Sequenced Read Archive PRJNA398089 and PRJNA400072. The source code for this work is available at GitHub (https://github.com/skimicrobe/GutNetMining).

## Abbreviations

IBD: Inflammatory bowel disease
ME/CFS: Myalgic encephalomyelitis/chronic fatigue syndrome
CRC: Colorectal cancer
CD: Crohn’s disease
UC: Ulcerative colitis
KOs: KEGG Orthologues
TCSs: Two-component system
Moco: Molybdenum cofactor
