# Supplementary material for: A novel computational approach for the mining of signature pathways using species co-occurrence networks in gut microbiomes
Source: BMC Microbiol. 2024 Nov 21;24(Suppl 1):490. doi: 10.1186/s12866-024-03633-6 (PMC11580338; doi:10.1186/s12866-024-03633-6)
Supplement: Supplementary file 2 — Supplementary Material 2. [file 12866_2024_3633_MOESM2_ESM.pdf]

A New Computational Approach for the Mining of Signature Pathways Using Species Co-occurrence Networks in Gut Microbiomes

Supplementary Material

Suyeon Kim, Ishwor Thapa, and Hesham Ali*

School of Interdisciplinary Informatics, University of Nebraska at Omaha, Omaha, NE 68182, USA

*To whom correspondence should be addressed.

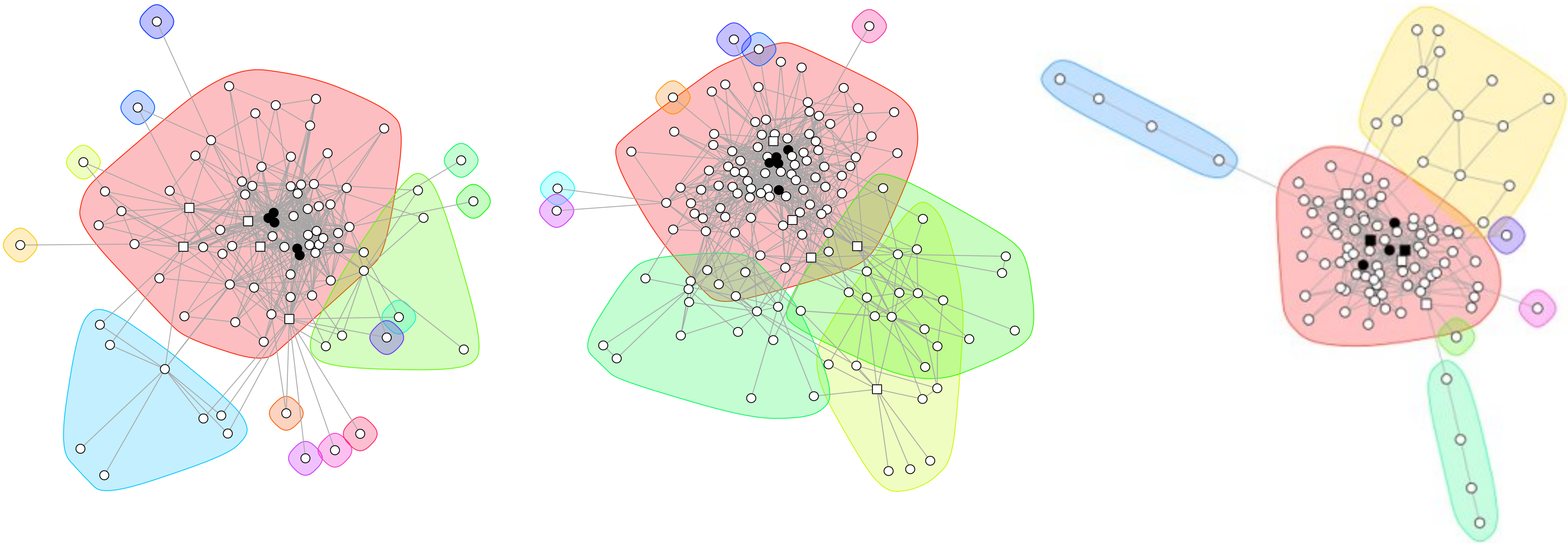

Figure S1: This figure illustrates how central species are captured differently at the community and the global level analysis. Each colored region represents a community. Each node (white circle) represents a different species of bacteria. Hub nodes are represented with a black circle, and betweenness nodes are represented with a white rectangle. (Left to Right: non-IBD control, CD, and UC from dataset 1).

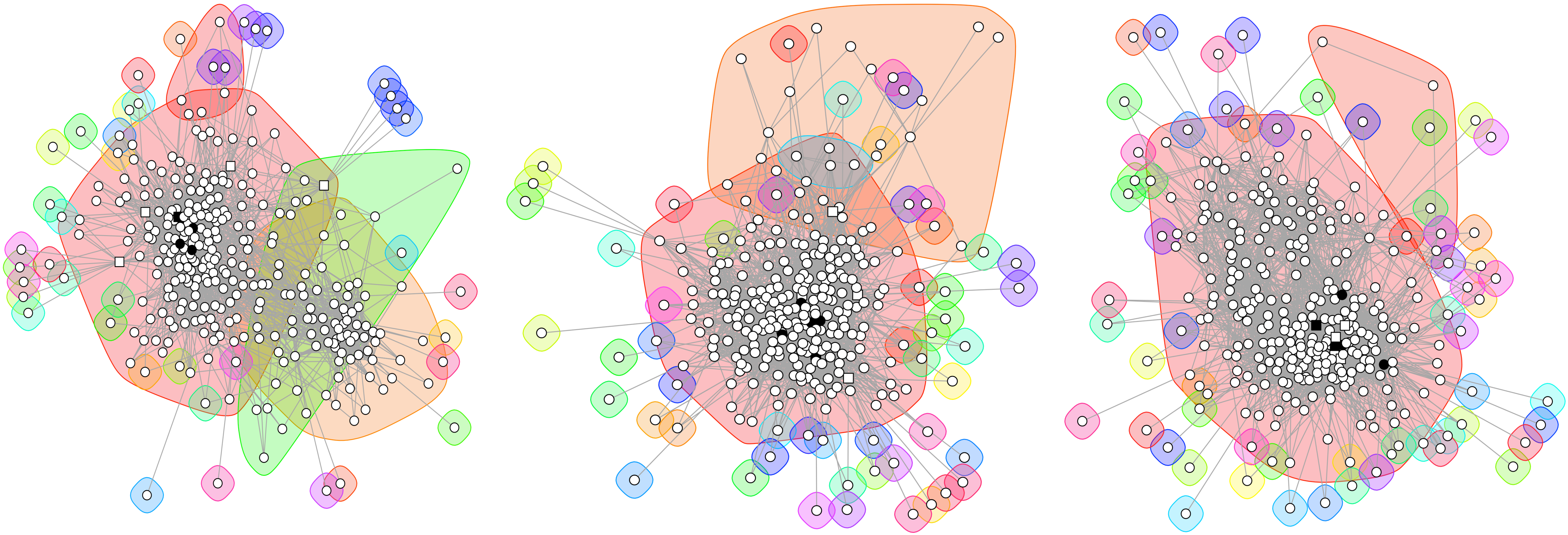

Figure S2: This figure illustrates how central species are captured differently at the community and the global level analysis. Each colored region represents a community. Each node (white circle) represents a different species of bacteria. Hub nodes are represented with a black circle, and betweenness nodes are represented with a white rectangle. (Left to Right: non-IBD control, CD, and UC from dataset 2).

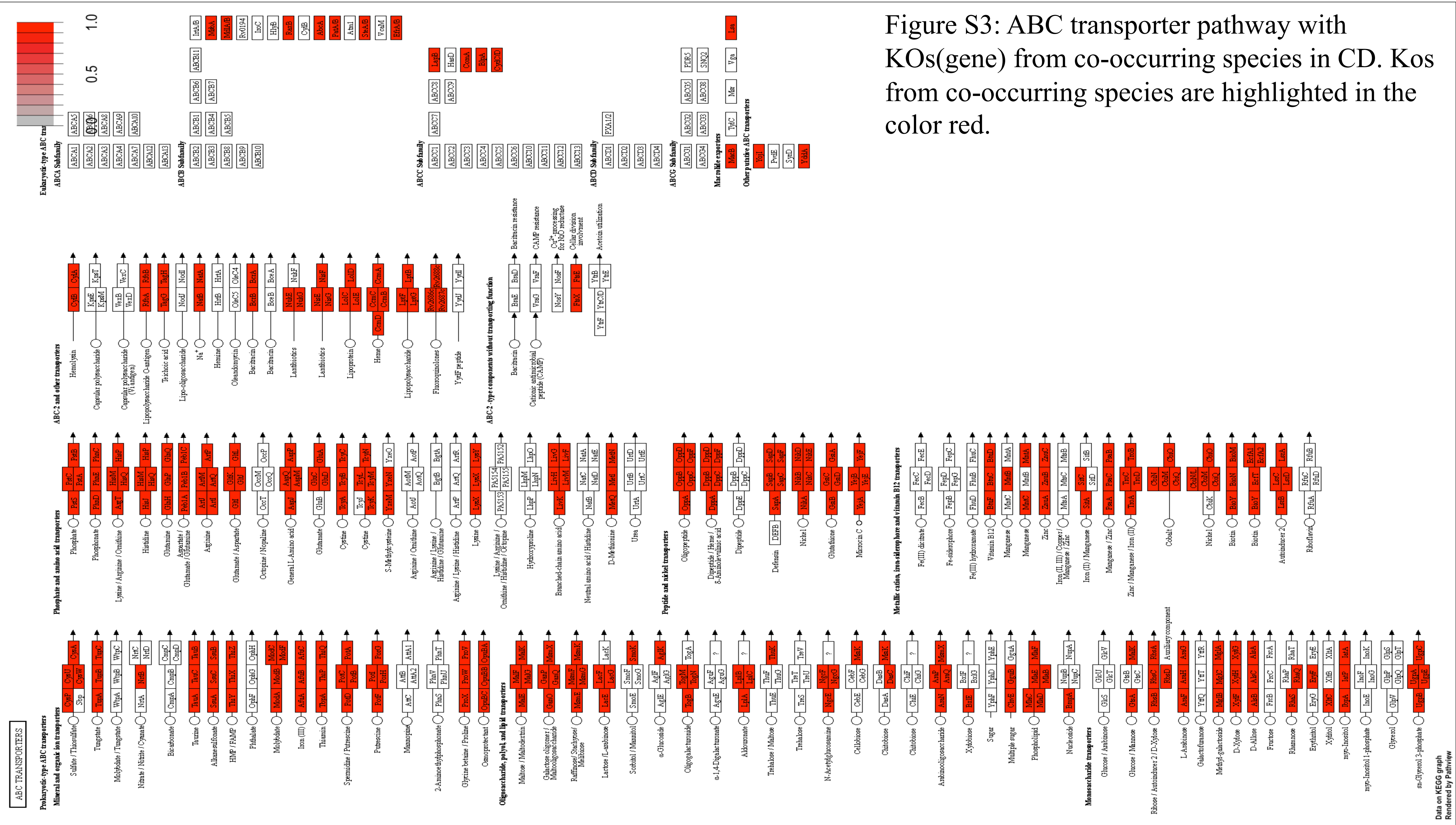

Figure S3: ABC transporter pathway with KOs(gene) from co-occurring species in CD. Kos from co-occurring species are highlighted in the color red.

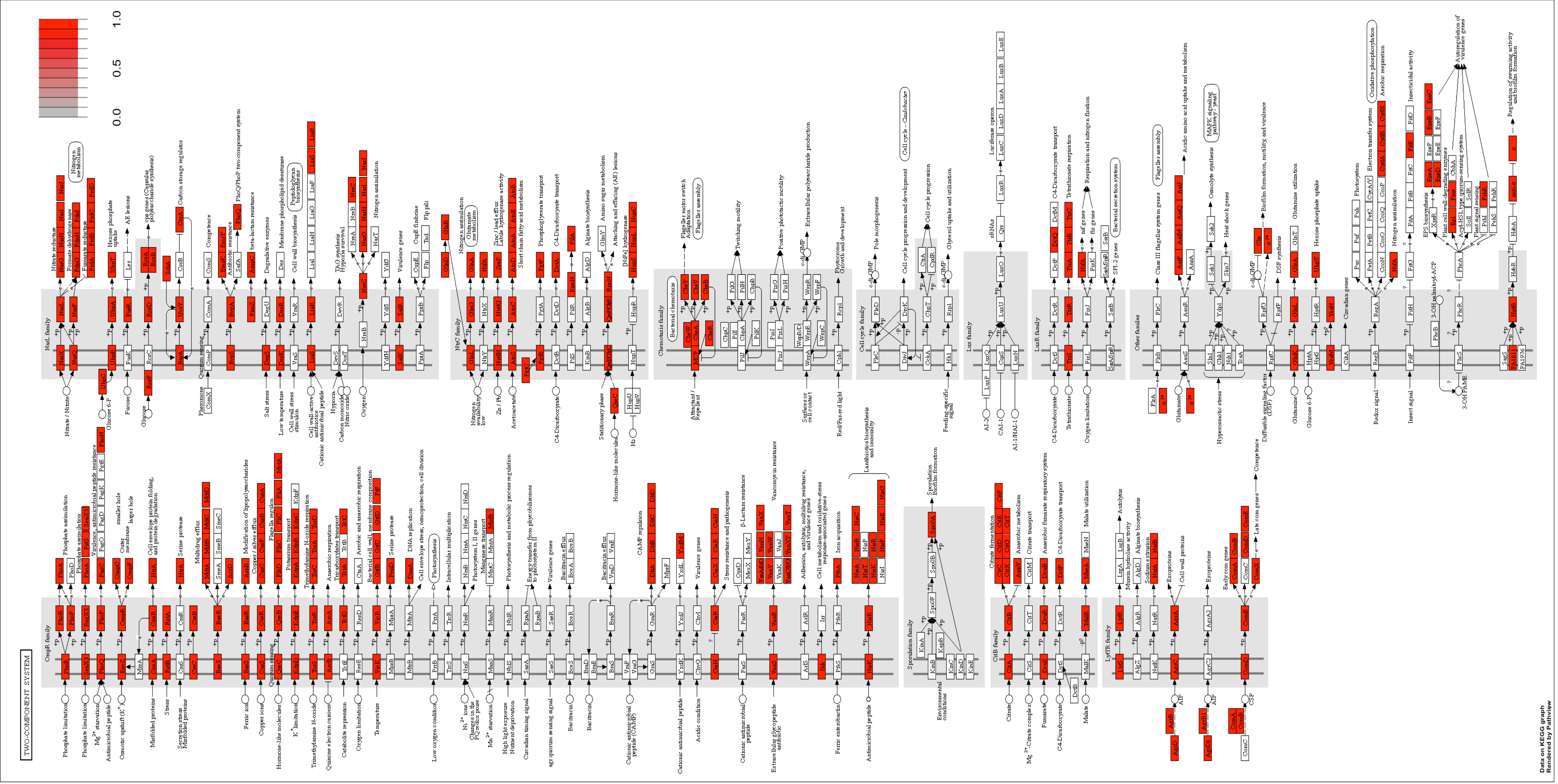

Figure S4: Two-component system pathway with KOs(gene) from co-occurring species in CD. Kos from co-occurring species are highlighted in the color red.

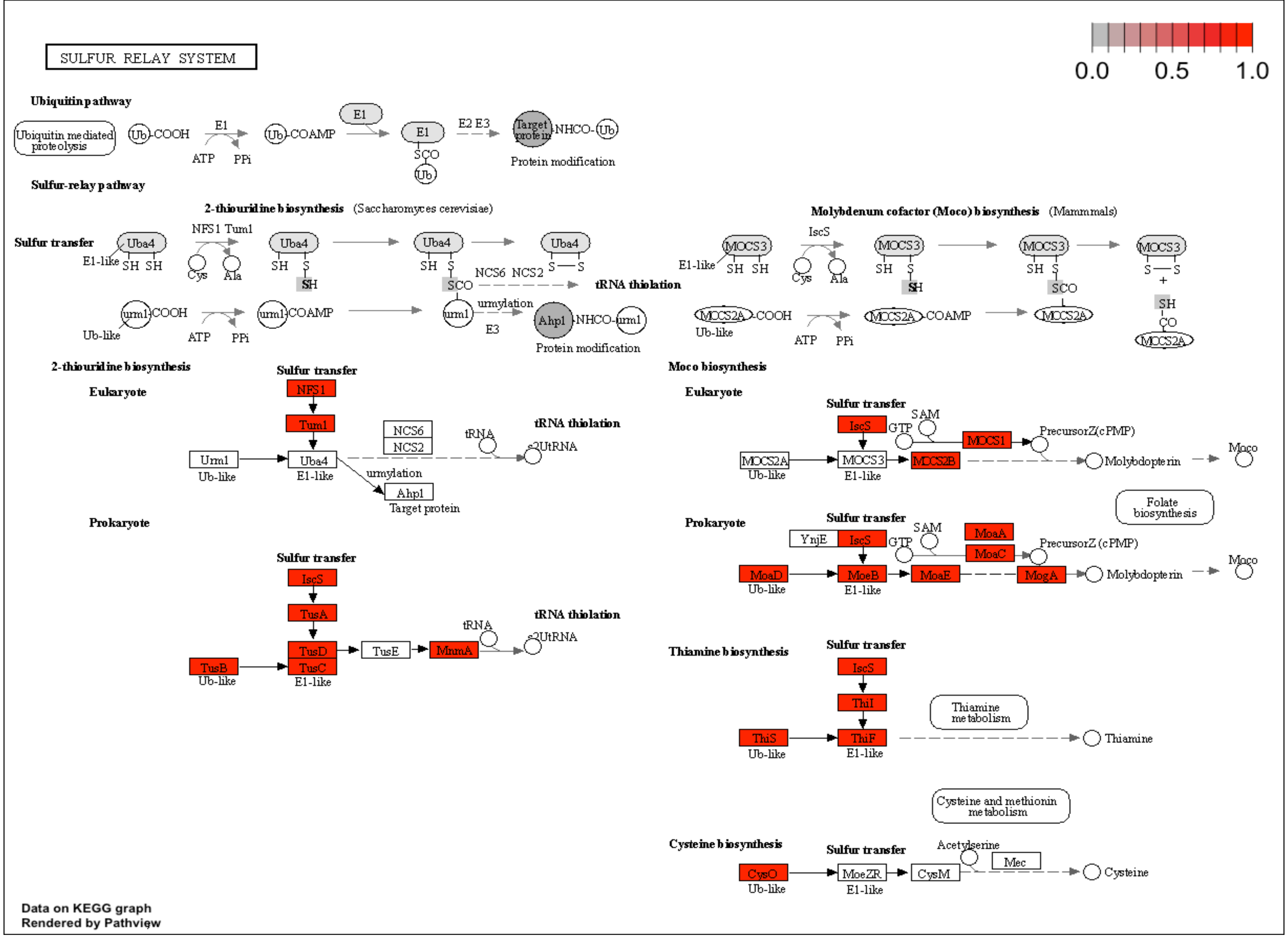

Figure S5: Sulfur relay system pathway with KOs(gene) from co-occurring species in UC.
